# Challenges of COVID‐19 Vaccination Effectiveness in Individuals with Alzheimer's Disease and Related Dementias

**DOI:** 10.1002/alz70860_099878

**Published:** 2025-12-23

**Authors:** Yijiong Yang

**Affiliations:** ^1^ Florida State University, Tallahassee, FL, USA

## Abstract

**Background:**

COVID‐19 vaccines became available in December 2020 to protect against severe acute respiratory syndrome coronavirus 2 (SARS‐CoV‐2). However, individuals with chronic conditions such as Alzheimer's disease and related dementias (ADRD) and mild cognitive impairment (MCI) face heightened risks of COVID‐19. These risks include increased vulnerability to COVID‐19‐related mortality, vaccine breakthrough infections, and long COVID symptoms. These risks are further exacerbated by challenges in adhering to preventive measures and managing clinical chronic conditions. Furthermore, the effectiveness of vaccines may decline over time, necessitating ongoing strategies to maintain protection.

**Method:**

A retrospective cross‐sectional study was conducted utilizing data from the All of Us Researcher Workbench. We extracted relevant data fields on participants’ demographic information, social determinants of health, and electronic health records (EHR). COVID‐19 vaccination data, including vaccination dates, vaccine brands and doses were collected from EHR drug records between December 14, 2020 and July 1, 2022. Vaccine effectiveness was assessed by examining the duration of immunity from the second dose of vaccination to reported COVID‐19 clinical conditions and laboratory results of SARS‐CoV‐2 (COVID‐19).

**Result:**

Fully vaccination rates were lower among Black and African American individuals with ADRD/MCI compared to non‐Hispanic Whites (82.7% vs 88.2%). Higher education and income were associated with higher vaccination rates in individuals with ADRD/MCI. However, at 6 months post vaccination, individuals with ADRD/MCI experienced significant waning of vaccine effectiveness compared to those without ADRD/MCI, with effectiveness decreasing from 96.7% two weeks after fully vaccination to 76.4% after 6 months, and 54.2% after 1 year and 6 months. Greater waning of vaccine effectiveness was observed in individuals aged over 65 years old, and multiple chronic conditions (e.g., COPD: 69.6%; Cancer: 73.5%; Stroke: 71.1%; Hypertension: 74.6%; Diabetes: 78.9%; Depression: 73.9%; Anxiety: 74.1%) at 6 months after fully vaccination compared to healthy adults.

**Conclusion:**

This study advances understanding of COVID‐19 vaccination effectiveness among individuals with ADRD/MCI and highlights the influence of chronic conditions on vaccine effectiveness. Efforts to promote vaccination boosters tailored to the currently circulating virus are essential for vulnerable people. Public health efforts can be focused on increasing booster shots among individuals with ADRD/MCI to ensure equitable and effective coverage.

